# Supplementary material for: Urothelial-lineage master transcription factor hub proteomics shows mechanisms impeding urothelial cancer cell differentiation
Source: bioRxiv. 2026 Aug 13:2026.08.12.744501. Preprint. [Version 1] doi: 10.64898/2026.08.12.744501 (PMC13640726; doi:10.64898/2026.08.12.744501)
Supplement: Supplementary file 1 [file media-1.pdf]

SUPPLEMENTARY MATERIAL

Table S1 (related to Figure 1) (Excel file). IP-LCMS/MS of IgG isotype control, FOXA1, CEBPB total spectral counts

Table S2 (related to Figure 1) (Excel file). Total spectral count comparison IP FOXA1 and CEBPB versus IP IgG isotype control

Table S3 (related to Figure 2). Umbrella cell and replication gene sets

Table S4 (related to Figure 2). Expression of umbrella and replication genes in primary UCs (TCGA RNA-seq data analyzed)

Table S5 (related to Figure 2). Gene expression by RNA-seq of Empty Vector vs ARID1A-transduced UC-6 cells (normalized counts)

Table S6 (related to Figure 2). Differentially expressed genes ARID1A transduction versus Empty Vector transduction (results of DESeq2 analyses)

2 supplementary figures

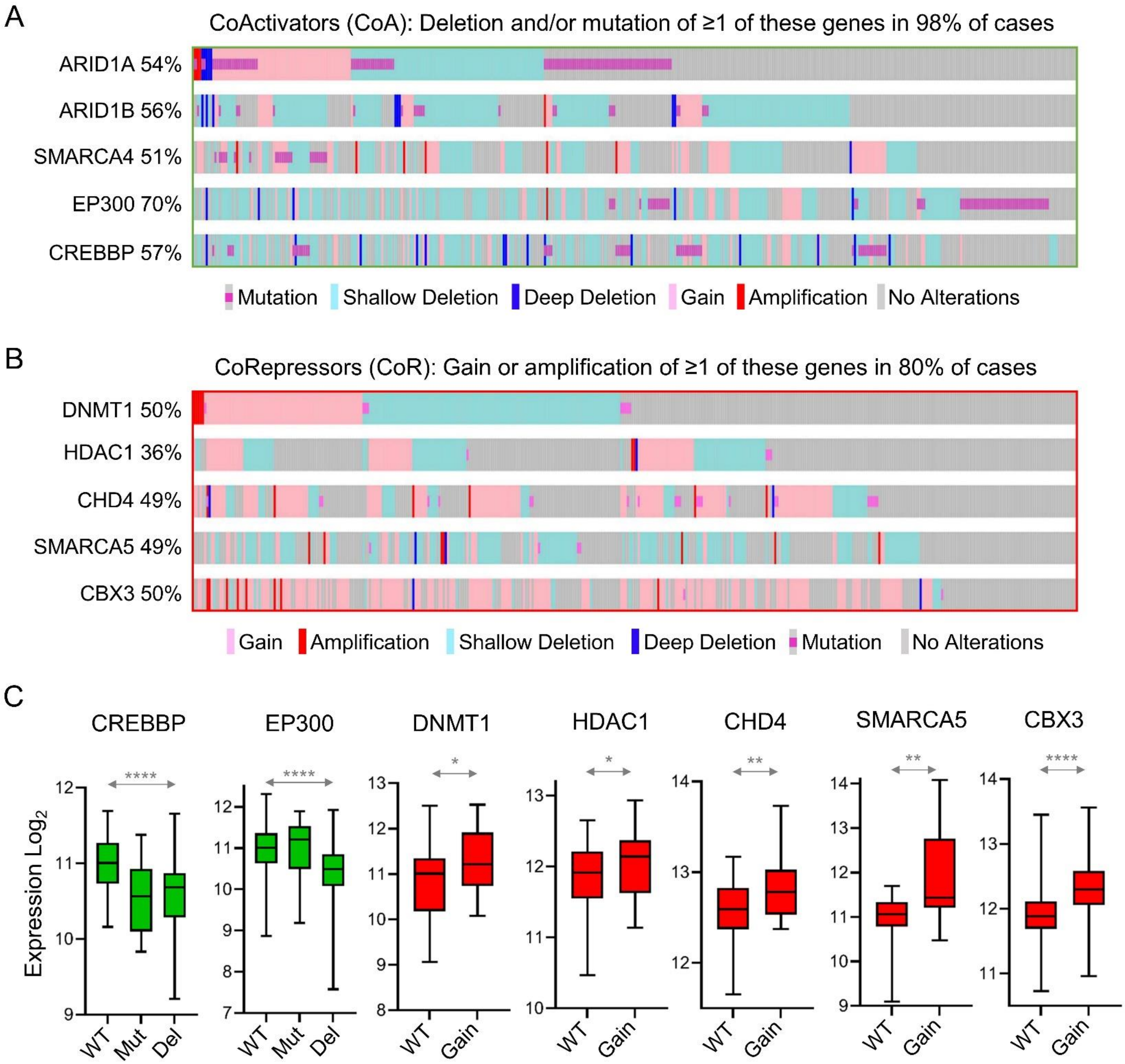

Figure S1. A) CoAs that write or read (as part of the SWI/SNF complex) the H3K27ac activation mark deleted or mutated in 98% of UCs (analyses of TCGA database[1]). These CoAs were prominent in the FOXA1/CEBPB interactomes in the IP-LCMS/MS analyses. Non-silent mutation and copy number calling as per TCGA[1], gene-level copy number estimates generated by the GISTIC2 method were thresholded to estimated values with -2 and -1 representing homozygous (deep) or single copy (shallow) deletion, 0 representing diploid normal copy, and 1 or 2 representing low-level (gain) or high-level copy number amplification. UCs n=397. **B) CoRs identified in the FOXA1/CEBPB interactomes were gained or amplified in 80% of UCs.** Analyses as per panel A. **C) Mutations and copy number deletions of CoA genes, and copy number gains and amplifications of CoR genes, in UCs decreased and increased expression of the genes respectively.** Copy number analyses as per panel A and B. Gene-level transcription estimates by RNA-Sequencing were analyzed as $log_2(x+1)$ transformed RSEM normalized counts. Median+/- interquartile range, Mann-Whitney test 2-sided, *p<0.05, **p<0.01, ***p<0.001, ****p<0.0001. UCs n=397.

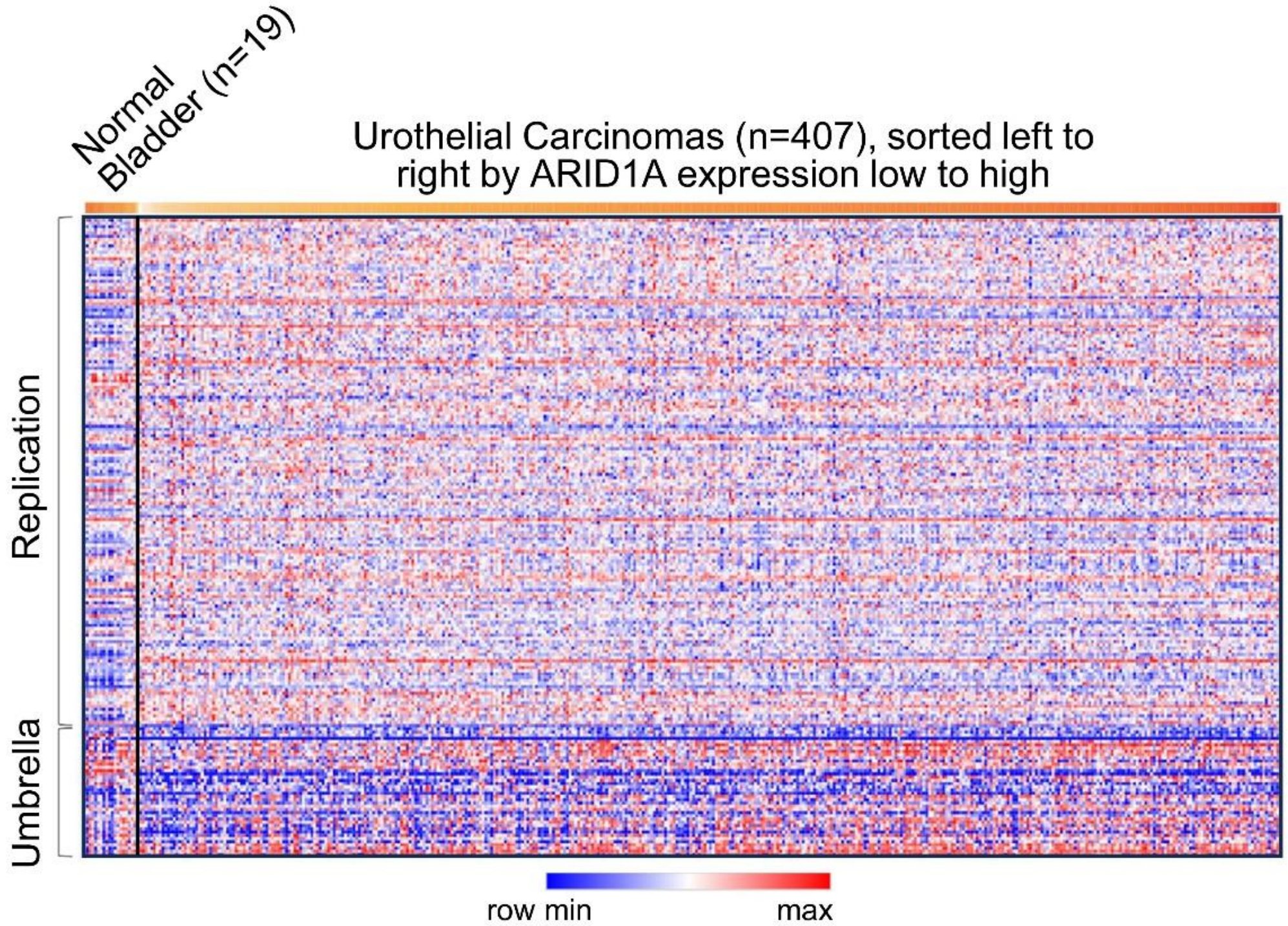

Figure S2. Umbrella gene expression was lower, and replication gene expression higher, in primary UC tissue compared to non-cancerous bladder tissue (TCGA RNA-sequencing, Table S4, primary UC samples n=407, non-cancer bladder n=19.

1. Cancer Genome Atlas Research N. Comprehensive molecular characterization of urothelial bladder carcinoma. *Nature*. 2014;507(7492):315-322.
